# Identification of a Metabolic Reaction Network from Time-Series Data of Metabolite Concentrations

**DOI:** 10.1371/journal.pone.0051212

**Published:** 2013-01-10

**Authors:** Kansuporn Sriyudthsak, Fumihide Shiraishi, Masami Yokota Hirai

**Affiliations:** 1 RIKEN Plant Science Center, Yokohama, Kanagawa, Japan; 2 JST, CREST, Kawaguchi, Saitama, Japan; 3 Graduate school of Bioresource and Bioenvironmental Sciences, Kyushu University, Fukuoka, Japan; National Institute of Environmental and Health Sciences, United States of America

## Abstract

Recent development of high-throughput analytical techniques has made it possible to qualitatively identify a number of metabolites simultaneously. Correlation and multivariate analyses such as principal component analysis have been widely used to analyse those data and evaluate correlations among the metabolic profiles. However, these analyses cannot simultaneously carry out identification of metabolic reaction networks and prediction of dynamic behaviour of metabolites in the networks. The present study, therefore, proposes a new approach consisting of a combination of statistical technique and mathematical modelling approach to identify and predict a probable metabolic reaction network from time-series data of metabolite concentrations and simultaneously construct its mathematical model. Firstly, regression functions are fitted to experimental data by the locally estimated scatter plot smoothing method. Secondly, the fitted result is analysed by the bivariate Granger causality test to determine which metabolites cause the change in other metabolite concentrations and remove less related metabolites. Thirdly, S-system equations are formed by using the remaining metabolites within the framework of biochemical systems theory. Finally, parameters including rate constants and kinetic orders are estimated by the Levenberg–Marquardt algorithm. The estimation is iterated by setting insignificant kinetic orders at zero, i.e., removing insignificant metabolites. Consequently, a reaction network structure is identified and its mathematical model is obtained. Our approach is validated using a generic inhibition and activation model and its practical application is tested using a simplified model of the glycolysis of *Lactococcus lactis* MG1363, for which actual time-series data of metabolite concentrations are available. The results indicate the usefulness of our approach and suggest a probable pathway for the production of lactate and acetate. The results also indicate that the approach pinpoints a probable strong inhibition of lactate on the glycolysis pathway.

## Introduction

Understanding metabolic pathways allows us to control metabolism, design a better metabolic system and optimise productivity. In vitro, in vivo and in silico research has been used to reconstruct the set of reactions that compose metabolic networks and their regulatory structure. However, it is still challenging to predict an unknown metabolic reaction network both experimentally and theoretically. For example, an *in vitro* experimental technique based on enzyme assays [1] can elucidate whether enzymes are inhibited or activated via interaction with metabolites, resulting in the clarification of a metabolic reaction network. However, this technique is costly, tedious and time-consuming because each enzyme activity needs to be measured individually in *in vitro* experimental systems specifically optimized for respective enzymes. Thus, it may be difficult to apply this technique to a large-scale metabolic system. On the other hand, time-dependent changes of metabolite concentrations can be determined *in vivo* [2] and a large amount of metabolomics data have been reported from the utilisation of high-throughput analytical instruments [3]. Canonical correlations and multivariate analysis are often used to analyse those metabolomics data. However, while correlations of metabolites have been successfully acquired, a network structure of the correlated metabolites remains unidentified.

Because of the experimental constraints, systems biology approaches are recently considered to be one of the alternatives for handling metabolomics data and analysing metabolic systems. Specifically, the mathematical modelling approaches have been exploited to analyse metabolic reaction networks [4]. In reality, however, information on metabolic reaction networks, metabolite concentrations and parameters such as rate constants and kinetic orders are required to construct an appropriate model. A well-known method is the utilisation of Michaelis–Menten type equations that express rates of enzymatic reactions [5]. However, it is not easy to identify each type of reaction because of *in vitro* experimental constraint mentioned above. Biochemical systems theory (BST) is an alternative method of analysing enzymatic reactions in network systems [6]–[8]. This theory provides a simple method for constructing a mathematical model once a network structure is available as a metabolic map, and it requires fewer parameters. Several techniques for estimating better parameter values have been proposed [9]–[11]. However, these techniques require a known metabolic reaction network for parameter estimation.

To overcome these difficulties, therefore, the present study explores a new approach for identifying a metabolic reaction network and simultaneously constructing a mathematical model. The approach consists of statistical and mathematical modelling techniques. The main concept of this approach is to employ time-series data to determine the structure of the metabolic reaction network. In principle, metabolites probably relate to others in a complicated network. A perturbation of a metabolite concentration causes changes in other metabolite concentrations. Thus, if changes in the time courses of metabolite concentrations are analysed, it becomes possible to predict and understand their metabolic reaction network. The present work therefore proposes such a new approach based on this idea.

## Results and Discussion

### Generic inhibition and activation model

**Figure 1:**
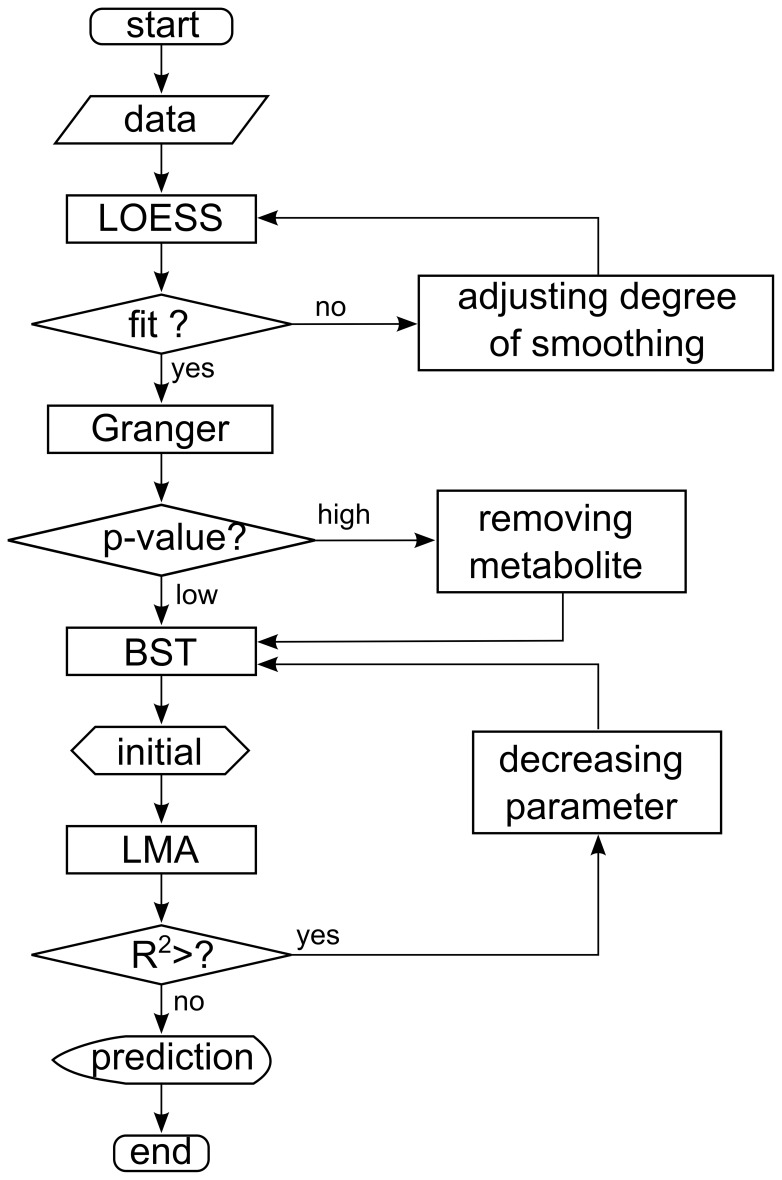
Proposed algorithm for metabolic reaction network identification.

The proposed algorithm is presented in Figure 1. As the real experimental data usually contain both biological and analytical errors, the analysis starts with smoothing noisy time-series data using locally estimated scatter plot smoothing (LOESS). Then, bivariate Granger causality is calculated to examine causal relationships between all pairs of metabolites, and unrelated metabolite pairs are removed from further consideration. A mathematical model is then formulated in S-system representation in the framework of biochemical systems theory (BST) by taking into consideration effects between all remaining metabolite pairs, followed by parameter estimation using nonlinear least-square method, namely Levenberg-Marquardt algorithm (LMA). The iterations from the mathematical modelling step to parameter estimation (BST to LMA) are simulated and a most insignificant metabolite is removed one by one in each iteration step. Finally, a probable metabolic network is identified.

**Figure 2:**
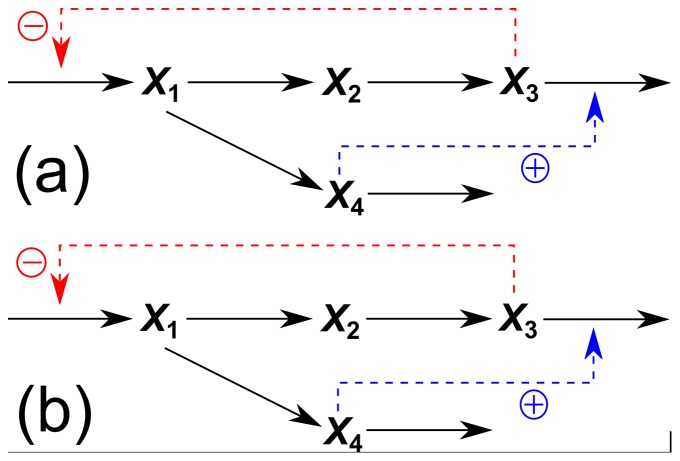
Real metabolic reaction network of the generic inhibition and activation model comparing with the predicted metabolic reaction network using our approach. (a) Real metabolic reaction network. (b) Predicted metabolic reaction network.

To validate if the algorithm is applicable, we start the study using a known metabolic reaction network, i.e., the generic inhibition and activation model (Figure 2A). This model has been widely employed as a metabolic case study in the development of parameter estimation techniques [12] because it imitates characteristics of a real metabolic pathway which includes a branching point and both inhibition and activation effects. Firstly, the time-series data for the metabolites X_1_–X_4_ were generated at 51 time points by using the mathematical model with parameter values described in equations 1–4. For the preliminary study, we consider a case without noise to properly evaluate the performance of the proposed approach. Therefore, the step of data smoothing by LOESS was not used.
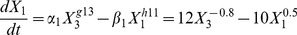
(1)

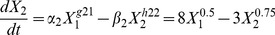
(2)

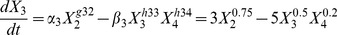
(3)

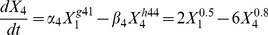
(4)where *X_i_* are metabolite concentrations. *α_i_* and *β_i_* are rate constants of net influxes and effluxes, and *g_ij_* and *h_ij_* are their kinetic orders.

**Figure 3:**
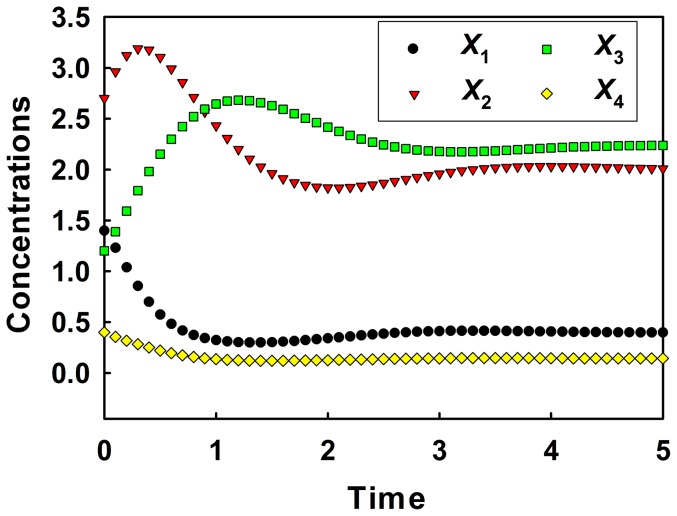
Time-series data of metabolite concentrations for the generic inhibition and activation model.

The time-series data *in silico* generated are plotted in Figure 3. The behaviour of metabolite concentrations is quite different to each other. It is therefore difficult to predict the relationship between the metabolite concentrations. To calculate correlation coefficient between metabolite concentrations, the normality distribution of each metabolite concentration was tested (data not shown). The result shows that the time-series data do not have normality (*p*-value<0.05), and the Spearman's rank correlation coefficient should be used to calculate the correlation coefficient. However, to broadly observe correlations among metabolite concentrations, we simply calculated both the Pearson's correlation coefficient and Spearman's rank correlation coefficient according to common methods for acquiring correlations of metabolite concentrations. The results, shown in Table S1, S2 in Information S1, indicate good positive and negative correlations between several metabolites. However, this finding does not indicate causal relationships between metabolites and effects of one metabolite on the other.

To obtain more information on network properties, the bivariate Granger causality test was executed to investigate relationships among metabolites. Table 1 tabulates the result of the bivariate Granger causality test for the generic inhibition and activation model. In theory, the Granger causality of a metabolite on itself cannot be calculated; hence, these data are not available. The result indicates that all p-values are much lower than a significance level of 0.01. This may be partly because we did not adjust the time lag (*u*) in equation 13 (see Methods) but retained its value as *u* = 1, implying that the present data point was used for predicting the value at the next time point. On the other hand, changes in the values of the time lag may have an effect on the Granger causality result. However, the p-values are still lower than the significance level of 0.01, although their value increases as the time lag increases (Table S3, S4, S5, S6, S7 in Information S1). Thus, we consider the time lag that maximises significance and set this lag to unity throughout the simulations. Only the data point at time *t*-1 was considered for predicting the value of the data point at time *t*. Furthermore, the Granger causality may give some false positive interactions if a network is very large, but it is not considered to be a serious problem here, since we perform this calculation only for finding the highest causality and removing unnecessary metabolites.

**Table 1:** Bivariate Granger causality test for the generic inhibition and activation model^a^.

	X_1_ = >	X_2_ = >	X_3_ = >	X_4_ = >
= >X_1_	N/A^b^	3.09E-22	5.21E-05	2.57E-17
= >X_2_	9.05E-44	N/A^b^	2.12E-26	2.54E-42
= >X_3_	1.27E-08	6.51E-58	N/A^b^	6.48E-16
= >X_4_	2.86E-26	5.07E-23	1.94E-15	N/A^b^

a Level of significance is 1% (p-values<0.01).

b N/A = not available.

From Table 1, it seems that each metabolite is Granger-caused by other metabolites. Hence, all metabolites must be considered in the next step calculation. The S-system equations were constructed and all metabolites were considered in the equations for both influxes and effluxes. It is possible to fit the metabolite concentrations using polynomial equations or sigmoidal curves and then calculate the slope values from the derivative of their equations. However, it should be noted that the concentrations are functions of time. This implies that even though one can calculate such slopes, these values may be different from their exact slope values directly calculated from S-system differential equations, because the exact slope values are functions of time and other metabolites. To make our approach practical, we calculated the slope values from neighbouring time-series data of metabolite concentrations. The differential equation for each metabolite was individually set as an objective function for parameter estimation.

The performance of LMA for estimating parameters in a well-known model was investigated before it is used in our algorithm. The results are given in Table S8 in Information S1. LMA finds only a local minimum, not a global one. It is therefore necessary to verify whether this non-linear regression method can successfully converge when power-law equations are used. Exact slope values from S-system equations were selected for this validation. The initial values for both rate constants and kinetic orders were set at unity. The results show that the parameters that converge using the exact slopes (estimated parameters b) are identical to their respective actual parameter values. This indicates that convergence behaviour of our parameter estimation procedure performs very well, especially for this system. In contrast, when the slopes were calculated from neighbouring data points, the converged parameter values (estimated parameters c) are slightly different from their true values. This is natural because these slopes were directly calculated and are not a function of other metabolites, unlike in the former case. In actual experiments, however, such exact slopes are not obtained and only the metabolite concentrations are available. Nevertheless, both sets of estimated parameters provide similar characteristics in terms of the behaviour of metabolite concentrations.

LMA provided fast convergences although the initial parameter values which were set to be unity are far from the true parameter values. The convergence times were calculated using GNU octave version 3.2.4 on Windows 7 platform with 2.93 GHz CPU. The convergence times of *X*_1_, *X*_2_, *X*_3_ and *X*_4_ with the exact slope values were 0.119, 0.176, 0.319 and 0.087 s, respectively, whereas those with the slope values calculated from neighbouring data were 0.120, 0.169, 0.382 and 0.090 s, respectively.

Once the performance of LMA was successfully elucidated, we exploited it to our algorithm. Assuming that the network is unknown, the S-system equations (equation 15) were set up and all parameters for all metabolites were primarily considered. Table 2 shows the first parameter iteration values obtained by LMA. It is clear that absolute values of some kinetic orders are very low compared with other parameters. The low absolute parameter values are considered to have little effect on the current system. The metabolites with such kinetic orders were thus removed one by one. New equations were re-organised and the parameter estimation by LMA was iterated. The results are shown in Information S2. Again, parameters quickly converged to their solutions. For the first iterations, the convergence times of *X*_1_, *X*_2_, *X*_3_ and *X*_4_ were 6.08, 11.9, 5.28 and 3.85 s, respectively. The convergence times also decreased with a decrease in the number of parameters.

**Table 2:** First iteration values for rate constants and kinetic orders in the generic inhibition and activation model.

	*X_i_*			
Parameters	*X*_1_	*X*_2_	*X*_3_	*X*_4_
*α_i_*	6.20321	7.37716	1.98302	0.34430
*g_i_*_1_	−0.36355	0.57390 *	0.01925	0.78879 *
*g_i_*_2_	−0.03741	−0.09305	0.87489 *	−0.17839
*g_i_*_3_	−1.37215 *	0.03478	−0.07383	0.02620
*g_i_*_4_	0.02696	0.00390	−0.05002	−0.56299
*β_i_*	3.89079	2.27677	3.76784	8.21555
*h_i_*_1_	0.82715 *	−0.03079	0.03747	−0.51013
*h_i_*_2_	−0.02735	0.81346 *	−0.13402	−0.25411
*h_i_*_3_	0.50948	0.04719	0.60945 *	0.25751
*h_i_*_4_	0.00431	0.01425	0.19157	1.74691 *
R^2^	1	1	1	1

* The significantly large kinetic orders are underlined.

The significantly large kinetic orders in Table 2 (more detail in Information S2) can be used to identify a metabolic reaction network. Although the metabolites with smaller kinetic orders may have some effect, the metabolites having large effects will probably be neighbouring metabolites in the metabolic pathway or metabolites that strongly inhibit or activate the metabolite of interest. Thus, the metabolites having large effects were selected for identification of an actual metabolic reaction network.

Figure 2B shows the metabolic reaction network identified from the converged results. The predicted network structure with the equations derived using our procedure is consistent with the original network structure in Figure 2A [9], [12]. A correct mathematical model and its parameters were also obtained simultaneously, as shown in Information S2. This suggests that our approach not only identifies a metabolic reaction network but also provides an appropriate mathematical model.

Although there is a constraint for using the bivariate Granger causality and also the parameter estimation using slopes may give slight calculation errors in the model construction, the above result clearly shows that our approach is theoretically consistent. Furthermore, it can provide a mathematical model for system analysis, although most of the systems biology approaches focus on either data analysis or model construction. On the other hand, actual experimental data contain biological and analytical errors and it may be difficult to obtain a large amount of time-series data. To evaluate the performance of our approach in practical application, therefore, the number of the time-series data for each metabolite concentration in the generic inhibition and activation model was decreased to 11 points and each data was allowed to include a noise in the range of 0–5% (Information S3). The result shows that it is still possible to estimate the metabolic reaction network if the time-series data set possesses clear characteristics and behaviours. It is therefore clear that our approach more depends on the quality of data than the quantity of data.

### Simplified model of glycolysis of *Lactococcus lactis*

We next discuss the glycolysis pathway of *Lactococcus lactis* because a number of metabolite concentrations have been reported for several types of micro-organisms genetically modified or perturbed both *in vitro* and *in vivo* [14]–[16]. The time-series data of metabolite concentrations for *Lactococcus lactis* MG1363 were taken from a number of studies [2], [17], [18].

According to these studies, several metabolite concentrations, such as phosphoenolpyruvate and phosphoglyceraldehyde, contain significant experimental errors, and it is difficult to validate the results. Consequently, these experimental data were neglected. In contrast, metabolites that have clear metabolic behaviours despite containing large experimental errors were considered here. The current system thus consists of five metabolites, including three extra- and two intra-cellular metabolites.

**Figure 4:**
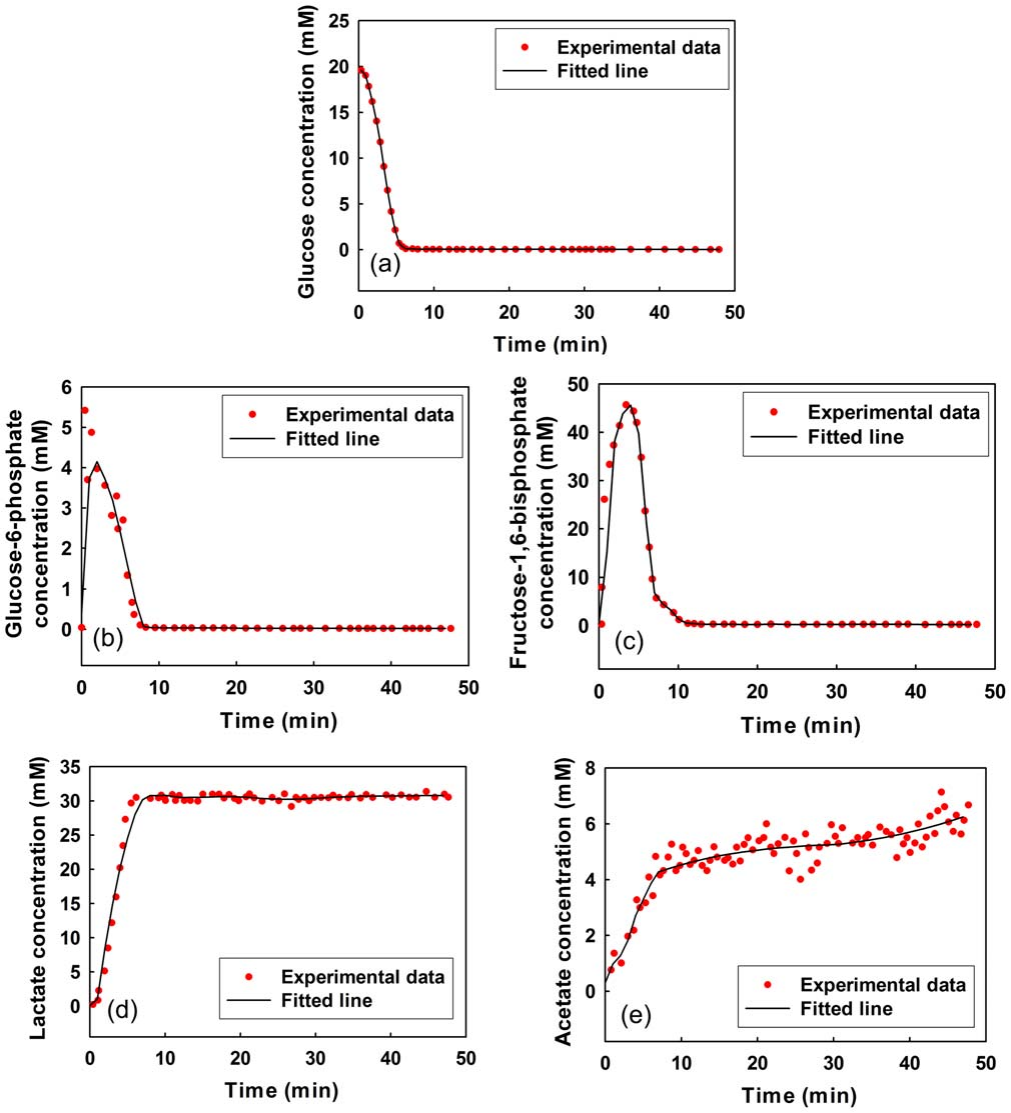
Time-series data of metabolite concentrations for the simplified model of glycolysis in Lactococcus lactis. (a) Glucose concentration. (b) Glucose-6-phosphate concentration. (c) Fructose-1,6-bisphosphate concentration. (d) Lactate concentration. (e) Acetate concentration.

We fitted the measured time-series data of metabolite concentrations obtained in Neves et al. [2], [17], [18] by LOESS. The parameters that control the degree of smoothing were arbitrarily adjusted (Table S18 in Information S4). The estimated time-series data of metabolite concentrations were produced from the results fitted by LOESS at time intervals of 1 min. Fifty-one data points for each metabolite concentration can be seen as lines in Figure 4.

The bivariate Granger causality for these estimated time-series data was calculated. The results are listed in Table 3. It is clear that some metabolites do not Granger-cause other metabolites (p-values>0.01) whereas others do. For instance, there exist high Granger-causes of *X*_2_ to *X*_1_, *X*_4_ to *X*_2_, *X*_1_ to *X*_3_, *X*_2_ to *X*_4_ and *X*_3_ to *X*_5_.

**Table 3:** Granger causality test for glycolysis pathway model^a^.

	*X*_1_ = >	*X*_2_ = >	*X*_3_ = >	*X*_4_ = >	*X*_5_ = >
= >*X*_1_	N/A^c^	2.70E-16^b^	1.09E-15	0.40 d	0.017 d
= >*X*_2_	7.38E-32	N/A^c^	2.38E-03	8.68E-37^b^	1.81E-12
= >*X*_3_	6.79E-18^b^	2.30E-15	N/A^c^	1.42E-15	7.36E-09
= >*X*_4_	0.028 d	3.89E-31^b^	1.01E-12	N/A^c^	0.046 d
= >*X*_5_	0.76 d	4.02E-04	5.40E-09^b^	0.95 d	N/A^c^

a Level of significance is 1% (p-values<0.01).

b Highest granger causality for each metabolites.

c N/A = not available.

d Insignificant Granger causalities are underlined.

**Figure 5:**
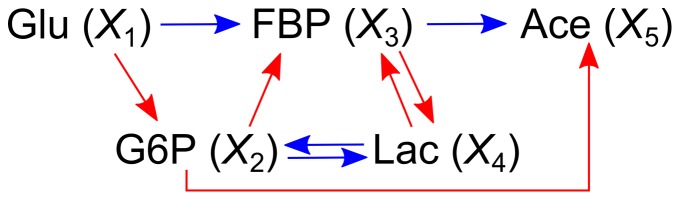
Probable metabolic reaction network of the glycolysis pathway in *Lactococcus lactis* predicted by Granger causality.

A procedure to construct a metabolic reaction by Granger causality is as follows. First, the influx to *X*_1_ (glucose) is not considered because it is the starting compound. Second, effluxes from *X*_4_ (lactate) and *X*_5_ (acetate) are also not considered because they are end products. Third, the metabolites that have insignificant Granger causalities are removed. Fourth, the metabolites having first and second Granger causalities are considered. As a result, it is possible to predict a pathway from the Granger causality, as illustrated in Figure 5, where the solid lines express the most significant causality for each metabolite and the broken lines express the second most significant causality. Although the Granger causality is useful for approximately understanding the metabolic network structure, it is still not enough to identify the actual structures because of insufficient information.

To more accurately predict the metabolic network structure, the mathematical modelling approach was repeatedly used right after the time-series data were statistically analysed. S-system equations (equation 15) were set up and parameters were estimated using LMA. In this case, the parameters for the metabolites which do not significantly Granger cause the other metabolites (underlined in Table 3) were removed or set to be zero. The remaining metabolites in the Granger causality test were then included in the influx terms of the S-system equations, whereas all metabolites were included in the efflux terms. The metabolites with the lowest kinetic order were removed one by one at each iteration step. Although a particular metabolite may have little effect on its efflux, it must be considered in the efflux term because the efflux is influenced by the metabolite. In addition, the metabolites with the highest Granger causality must be considered in the entire calculation because they are statistically significant.

Table 4 presents the parameters that were determined by LMA in the glycolysis model and the predicted model (equations 5–9) are described as follows:
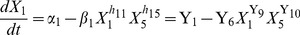
(5)

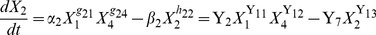
(6)

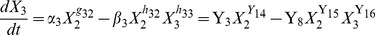
(7)

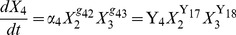
(8)

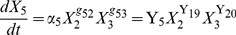
(9)where *X_i_* are metabolite concentrations. *α_i_* and *β_i_* are rate constants of total influxes and effluxes, respectively, and *g_ij_* and *h_ij_* are their kinetic orders. Y*_i_* represent unknown parameters assigned for both rate constants and kinetic orders.

**Table 4:** Parameters determined by LMA of the simplified model of the glycolysis pathway of *Lactococcus lactis*.

	*X_i_*				
Parameters	*X*_1_	*X*_2_	*X*_3_	*X*_4_	*X*_5_
*α_i_*	0.141 [Y_1_]	2.36 [Y_2_]	98.9 [Y_3_]	0.671 [Y_4_]	0.216 [Y_5_]
*g_ij_*		0.199 [Y_11_]	1.14 [Y_14_]	1.15 [Y_17_]	0.324 [Y_19_]
*g_ij_*		−0.317 [Y_12_]		0.199 [Y_18_]	0.183 [Y_20_]
*β_i_*	0.191 [Y_6_]	1.373 [Y_7_]	88.7 [Y_8_]		
*h_ij_*	0.943 [Y_9_]	0.329 [Y_13_]	0.993 [Y_15_]		
*h_ij_*	1.68 [Y_10_]		0.0773 [Y_16_]		
R^2^	0.9996	0.9987	0.9486	0.9816	0.9198

Y*_k_* are parameter values for both rate constants and kinetic orders (*k*  = 1, 2, 3,…, 20) are parameter values for both rate constants and kinetic orders.

Table 4 also includes the R-squared values in each removal process. As the number of iterations increases, the R-squared value usually decreases from unity (Information S4). A low R-squared value implies that the values calculated using the reconstructed equations do not fully agree with the experimental data. This is natural because the degree of fitness is lowered as a result of the reduction in the number of parameters.

**Figure 6:**
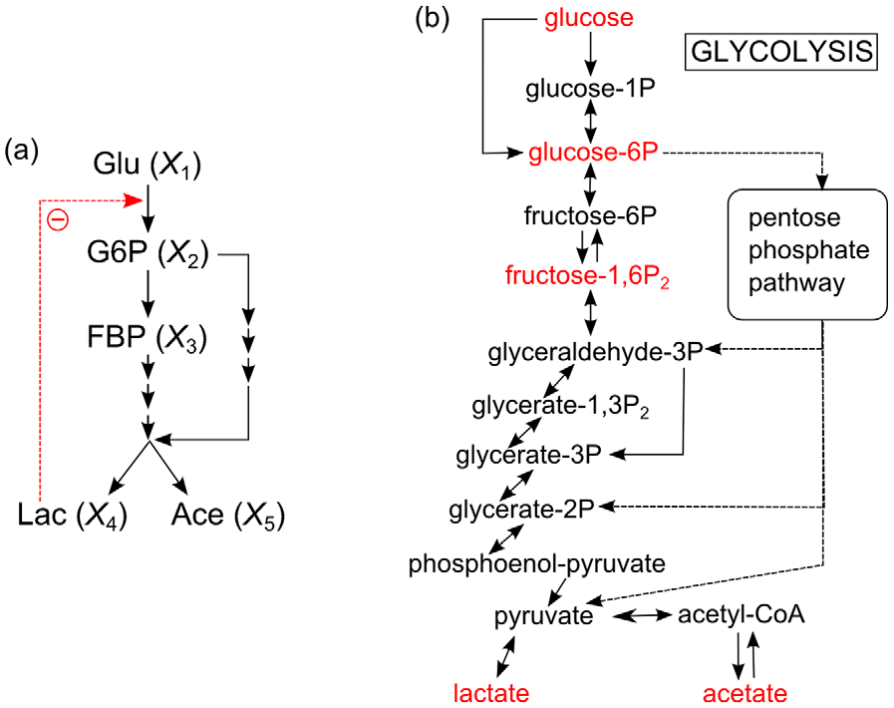
Comparison of metabolic reaction networks of the glycolysis pathway in Lactococcus lactis predicted by our approach and taken from KEGG. (a) illustrates the pathway predicted by our approach whereas (b) illustrates the pathway taken from KEGG and the red characters in Figure 6B indicate metabolites considered in our prediction model.

Figure 6A shows the metabolic reaction network predicted using the remaining parameters. Glucose (Glu) is converted to glucose-6-phosphate (G6P), which is successively converted to fructose-1,6-bisphosphate (FBP). This agrees with the structure of the actual glycolysis pathway acquired from KEGG (Figure 6B). Interestingly, our approach suggests that G6P has a pathway, allowing it to be converted to lactate (Lac) and acetate (Ace). This pathway could be regarded as the part of pentose phosphate pathway, although the flux through this pathway is not high [19]. Our approach further suggests that Lac strongly inhibits the formation of G6P. This interaction is related to the inhibition of acids on cells [20].

**Figure 7:**
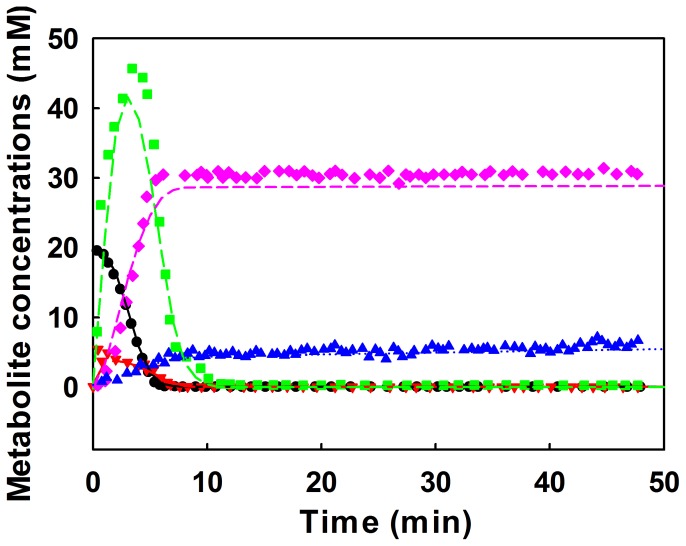
Comparison of values calculated by a constructed mathematical model and experimental data (black circle-Glu data, red down-pointing triangle-G6P data, green square-FBP data, purple diamond-Lac data, blue up-pointing triangle-Ace data and simulations for Glu, G6P, FBP, Lac and Ace are in black line, red line, green line, pink line and blue line, respectively).

Identification of the probable metabolic reaction network automatically leads to the formulation of a mathematical model in the S-system. As shown in Figure 7, the values calculated by the mathematical model are in agreement with the experimental ones, implying that our approach has good performance.

To further verify whether the mathematical model is appropriate, we calculated the instantaneous bottleneck ranking (BR) indicator defined as
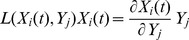
(10)


This indicator is a product of the logarithmic gain *L*(*X_i_*,*Y_j_*) and the metabolite concentration *X_i_* and provides the time-transient response of the dependent variable *X_i_* to an infinitesimal percentage change in the independent variable *Y_j_* [21]. A positive value of the instantaneous BR indicator indicates that an increase in an enzyme activity increases the relevant metabolite concentration from its initial concentration, whereas a negative value indicates that an increase in the relevant enzyme activity decreases the relevant metabolite concentration.

**Figure 8:**
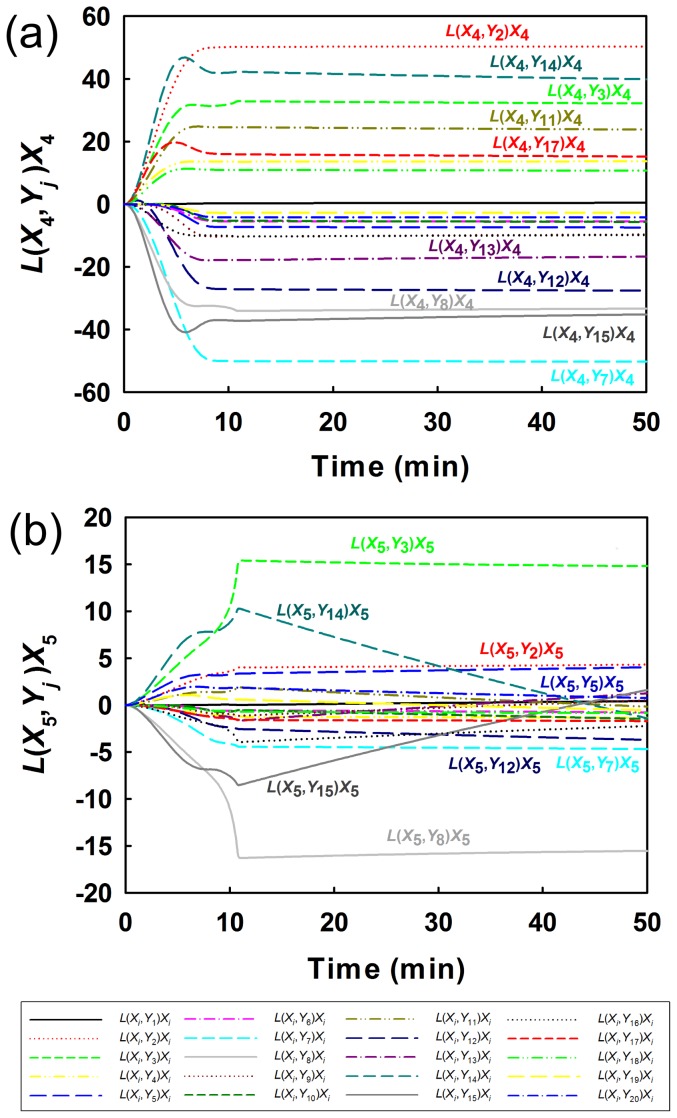
Instantaneous bottleneck ranking indicators for the predicted glycolysis pathway using our approach. (a) is L(X_4_,Y_j_)X_4_ for lactate whereas (b) is L(X_5_,Y_j_)X_5_ for acetate.

Figure 8 shows the time courses of the instantaneous BR indicators for lactate (X_4_) and acetate (X_5_) after the individual rate constants and kinetic orders (Table 4) increases at *t* = 0 (additional information is available in Table S24 in Information S4). Overall, the BR indicators for the lactate concentration increase or decrease more significantly than do those for the acetate concentration, suggesting that lactate is more easily formed than acetate. The difficulty in the formation of acetate arises because the flux for lactate formation is higher than that for acetate formation. Moreover, ranking of enzymes based on the BR indicators reveals that the bottleneck enzyme for lactate formation is Y_2_ when it is increased and Y_7_ when it is decreased, while that for acetate formation is Y_3_ when it is increased and Y_8_ when it is decreased. These finding are supported by the previously reported experimental data [22]. Thus, the analytical results using the instantaneous BR indicators indirectly support the reliability of our network identification approach.

Unlike other statistical approaches using correlation or causality, our approach can not only identify a metabolic reaction network but also provide a mathematical model simultaneously. Furthermore, it provides kinetic parameters which allows us to straightforwardly analyse the metabolic system using the obtained mathematical model.

## Conclusions

The present study investigated an approach to identify a metabolic reaction network structure from time-series data of metabolite concentrations and simultaneously obtain its mathematical model in the S-system equations within the framework of BST. The Granger causality test was used to statistically identify interactions among metabolites and then remove metabolites which have insignificant causality to the considered metabolite. This result was used to form a mathematical model in the S-system representation. The parameters, namely, rate constants and kinetic orders in this mathematical model, were estimated by the Levenberg–Marquardt method. This estimation process was iterated to remove the least significant metabolite of each total influx and efflux according to the magnitudes of the kinetic orders. Consequently, the final form of the mathematical model was used to predict a probable structure for the metabolic reaction network system. A series of theoretical analyses clearly show that our approach is effective in identifying a metabolic reaction network. In the future, an in vitro experiment for measuring individual enzyme activities may also be performed on the basis of the prediction to reconstruct a newly possible metabolic pathway.

## Methods

To efficiently identify a metabolic reaction network using time-series data of metabolite concentrations, we use statistical and mathematical modelling techniques as described below.

### Locally estimated scatter plot smoothing

The locally estimated scatter plot smoothing (LOESS) method is a non-parametric statistic which does not require any specific function to fit a mathematical model. Hence, it is very flexible in fitting experimental data containing noise or experimental errors. The regression function is locally approximated by the value of a function in some specified parametric class [23]. Such a local approximation is obtained by fitting a regression surface to data points within a chosen neighbourhood of the point:
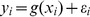
(11)where *y_i_* is the *i*th measurement of the response *y*, *x_i_* is the corresponding measurement of predictors, *g* is the regression or the smooth function and *ε_i_* is the random error. The weights are given by the tricube function:
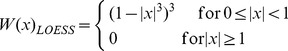
(12)


The value of weight function is low when *x_k_* is distant from *x_i_*. If its value is increased, the influence from the data points located in the neighbourhood will be increased. This results in increased smoothness of the smoothed points. A piecewise function is used to handle the data that cannot be properly fitted.

### Bivariate Granger causality

The Granger causality test is a statistical hypothesis test used to determine whether one time series causes another. It is widely used in economics and has recently been employed to integrate omics data, i.e. transciptomics and metabolomics [13]. The present study introduces this test to evaluate causality among metabolites. Direct relationships between two metabolites were evaluated using the bivariate Granger causality test [24] on the basis of the following equations: 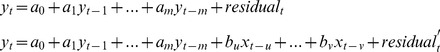
(13)where *x* and *y* are the stationary time series for testing the null hypothesis that *x* does not Granger-cause *y*. Appropriate lagged values of *y* are found and included in a univariate autoregression of *y*. The symbol m denotes the largest lag length for which the lagged dependent variable is significant, *u* is the shortest lag length and *v* is the longest length for which the lagged value of *x* is significant. The time lag was set to be unity, denoting that the value at the present data point was used to predict the value at the next time point.

An F-test for equality of variances is used to verify whether the residuals are significant. An index measuring the strength of the causal interaction is defined as
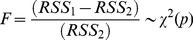
(14)where *RSS*_1_ and *RSS*_2_ are the sums of squared residuals of *residual_t_* and *residual_t_*', respectively.

The null hypothesis is rejected if the F calculated from the data is greater than the critical value of the F distribution for some desired false rejection probability; the present study used 0.01 for the significant value.

### Biochemical systems theory (BST)

Biochemical systems theory (BST) provides a powerful procedure for characterising biochemical systems [6]–[8]. BST describes non-linear systems in terms of power-law functions. The present study uses the S-system representation within the framework of BST:
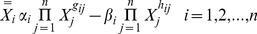
(15)where *X_i_* (*i* = 1,…, *n*) are the metabolite concentrations for *n* dependent variables and *α_i_* and *β_i_* are the rate constants of influxes and effluxes, while *g_ij_* (*j* = 1,…, *n*) and *h_ij_* (*j* = 1,…, *n*) are kinetic orders of influxes and effluxes, respectively. The S-system describes a metabolic reaction network by individually aggregating influxes and effluxes for each metabolite pool, reducing the number of parameters.

### Levenberg–Marquardt algorithm

Non-linear least-squares methods use parameter estimation iterations to reduce the sum of the squared errors between each function value and a measured data point. LMA is a combination of the gradient descent method and the Gauss–Newton method [25]–[27].

The chi-squared error criterion is given as
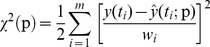
(16)where *y*(*t_i_*) is the measured value, *y*(*t_i_*;p) is the curve fitting function and *w_i_* is the measure of error in measurement *y*(*t_i_*).

The gradient of the chi-squared objective function with respect to the parameters is given as follows:
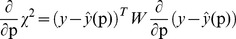
(17)where the weighting matrix *W* is diagonal with *W* = 1/*w_i_* ^2^

The Gauss–Newton method denotes the perturbed model parameters that are locally approximated by first-order Taylor series expansion as
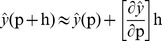
(18)


The Levenberg algorithm adaptively varies and updates the parameters between the gradient descent method and Gauss–Newton as
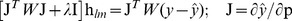
(19)where *λ* represents the algorithm parameter.

LMA implemented here uses the following modified equation [28]:
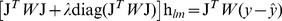
(20)


Small values of *λ* lead to a Gauss–Newton update, whereas large values of *λ* lead to a gradient descent update.

### Proposed algorithm

Our proposed algorithm is illustrated in Figure 1. The calculation starts with data smoothing by the LOESS method, followed by using the Granger causality test for removing an unnecessary metabolite and then estimating parameters in the S-system equations by LMA. In the iteration of this series of methods, parameters having the least effect are removed one by one under the criterion that each term in the equation has at least one metabolite or the R-squared value does not remarkably decrease within a satisfactory degree of fitness.

### Case studies

Two mathematical models were used in the present study. One is the generic inhibition and activation model [9]–[12], which is well known and useful in validating our approach. The other is the simplified model of glycolysis of *Lactococcus lactis* MG1363 [17], [29]–[31]. Actual experimental data are available for this model and it is therefore useful for verifying whether our approach is practical.

## Supporting Information

Information S1 **Evaluation of correlations, bivariate Granger causality and Levenberg-Marquardt Algorithm (LMA) performance.**
(DOC)

Information S2 **Additional information for the generic inhibition and activation model.**
(DOC)

Information S3 **Evaluation of the performance of our approach in practical application.**
(DOC)

Information S4 **Additional information for the** ***Lactococcus lactis*** **model.**
(DOC)
