# Supplementary material for: Identification of a Metabolic Reaction Network from Time-Series Data of Metabolite Concentrations
Source: PLoS One. 2013 Jan 10;8(1):e51212. doi: 10.1371/journal.pone.0051212 (PMC3542379; doi:10.1371/journal.pone.0051212)
Supplement: Information S1 — Evaluation of correlations, bivariate Granger causality and Levenberg-Marquardt Algorithm (LMA) performance. (DOC) [file pone.0051212.s001.doc]

**Supporting Information S1:**

**Evaluation of correlations, bivariate Granger causality and Levenberg- Marquardt Algorithm (LMA) performance**

**Table S1.** Spearman's rank correlation coefficient between metabolite concentrations for the generic inhibition and activation model

|  | *X*_1_ | *X*_2_ | *X*_3_ | *X*_4_ |
| --- | --- | --- | --- | --- |
| *X*_1_ | 1.0 | 0.44683 | -0.90079 | 0.922305 |
| *X*_2_ | 0.44683 | 1.0 | -0.21947 | 0.650544 |
| *X*_3_ | -0.90079 | -0.21947 | 1.0 | -0.76435 |
| *X*_4_ | 0.922305 | 0.650544 | -0.76435 | 1.0 |

**Table S2.** Pearson's correlation coefficient between metabolite concentrations for the generic inhibition and activation model

|  | *X*_1_ | *X*_2_ | *X*_3_ | *X*_4_ |
| --- | --- | --- | --- | --- |
| *X*_1_ | 1.0 | 0.650968 | -0.90284 | 0.98692 |
| *X*_2_ | 0.650968 | 1.0 | -0.42815 | 0.761395 |
| *X*_3_ | -0.90284 | -0.42815 | 1.0 | -0.85832 |
| *X*_4_ | 0.98692 | 0.761395 | -0.85832 | 1.0 |

Table S3-S7 tabulates bivariate Granger causality test for the generic inhibition and activation model at the time lag *u* = 2, 3, 4, 5 and 10, respectively. The most significant p-values for each case are indicated in red. The details are also described in the paper.

**Table S3.** Bivariate Granger causality test for the generic inhibition and activation model at the time lag *u* = 2

|  | X_1_=> | X_2_=> | X_3_=> | X_4_=> |
| --- | --- | --- | --- | --- |
| => X_1_ | N/A | 4.85E-50 | 8.40E-39 | 2.20E-33 |
| => X_2_ | 1.90E-31 | N/A | 1.74E-15 | 0.01674 |
| => X_3_ | 3.56E-30 | 1.09E-24 | N/A | 8.23E-33 |
| => X_4_ | 1.00E-45 | 1.58E-06 | 0.000249 | N/A |

**Table S4.** Bivariate Granger causality test for the generic inhibition and activation model at the time lag *u* = 3

|  | X_1_=> | X_2_=> | X_3_=> | X_4_=> |
| --- | --- | --- | --- | --- |
| => X_1_ | N/A | 3.24E-61 | 7.33E-31 | 1.58E-43 |
| => X_2_ | 3.21E-24 | N/A | 1.89E-24 | 1.04E-24 |
| => X_3_ | 5.73E-19 | 5.15E-18 | N/A | 3.86E-18 |
| => X_4_ | 3.14E-43 | 7.68E-45 | 2.13E-43 | N/A |

**Table S5.** Bivariate Granger causality test for the generic inhibition and activation model at the time lag *u* = 4

|  | X_1_=> | X_2_=> | X_3_=> | X_4_=> |
| --- | --- | --- | --- | --- |
| => X_1_ | N/A | 9.94E-24 | 4.90E-28 | 1.59E-28 |
| => X_2_ | 0.116349 | N/A | 0.060223 | 0.047614 |
| => X_3_ | 2.43E-05 | 0.000211 | N/A | 8.11E-05 |
| => X_4_ | 7.34E-20 | 7.35E-18 | 2.67E-15 | N/A |

**Table S6.** Bivariate Granger causality test for the generic inhibition and activation model at the time lag *u* = 5

|  | X_1_=> | X_2_=> | X_3_=> | X_4_=> |
| --- | --- | --- | --- | --- |
| => X_1_ | N/A | 2.07E-13 | 1.63E-12 | 2.49E-14 |
| => X_2_ | 0.863745 | N/A | 0.358756 | 0.41525 |
| => X_3_ | 0.000382 | 0.000672 | N/A | 0.000259 |
| => X_4_ | 0.001776 | 0.00166 | 0.001145 | N/A |

**Table S7.** Bivariate Granger causality test for the generic inhibition and activation model at the time lag *u* = 10

|  | X_1_=> | X_2_=> | X_3_=> | X_4_=> |
| --- | --- | --- | --- | --- |
| => X_1_ | N/A | 0.526197 | 0.809429 | 0.349787 |
| => X_2_ | 0.604804 | N/A | 0.652208 | 0.809074 |
| => X_3_ | 0.549409 | 0.330634 | N/A | 0.540083 |
| => X_4_ | 0.44226 | 0.790581 | 0.887279 | N/A |

**Table S8.**Rate constants and kinetic orders for the generic inhibition and activation model (a) actual parameters, (b) estimated parameters from true slopes, (c) estimated parameter from direct calculation for slopes

| Parameters | Actual parameters (a) | Estimated parameters (b) | Estimated parameters (c) |
| --- | --- | --- | --- |
| *α*_1_  *α*_2_  *α*_3_  *α*_4_  *β*_1_  *β*_2_  *β*_3_  *β*_4_ | 12.0  8.0  3.0  2.0  10.0  3.0  5.0  6.0 | 12.00000  8.00000  3.00000  2.00000  10.00000  3.00000  5.00000  6.00000 | 11.71895  7.90009  3.12558  2.08017  9.73394  2.90176  4.97183  5.89774 |
| *g*_13_  *h*_11_  *g*_21_  *h*_22_  *g*_32_  *h*_33_  *h*_34_  *g*_41_  *h*_44_ | -0.8  0.5  0.5  0.75  0.75  0.5  0.2  0.5  0.8 | -0.80000  0.50000  0.50000  0.75000  0.75000  0.50000  0.20000  0.50000  0.80000 | -0.81521  0.50982  0.50829  0.76902  0.72239  0.47047  0.17377  0.47096  0.75724 |
