# Supplementary material for: Identification of a Metabolic Reaction Network from Time-Series Data of Metabolite Concentrations
Source: PLoS One. 2013 Jan 10;8(1):e51212. doi: 10.1371/journal.pone.0051212 (PMC3542379; doi:10.1371/journal.pone.0051212)
Supplement: Information S2 — Additional information for the generic inhibition and activation model. (DOC) [file pone.0051212.s002.doc]

**Supporting Information S2:**

**Additional information for the generic inhibition and activation model**

Table S9-S12 tabulates the iterations for parameter values of *X*_1_ to *X*_4_, respectively. The estimation loops were iterated until the most suitable parameters (indicated in red) were found as described in detail in the paper.

**Table S9.** Parameter values of *X*_1_ for each iteration using LMA

| Iteration | 1 | 2 | 3 | 4 | 5 | 6 | 7 |
| --- | --- | --- | --- | --- | --- | --- | --- |
| *α*_1_ | 6.20321 | 5.97043 | 5.12574 | 3.76481 | 3.82485 | 11.66166 | 11.71895 |
| *g*_11_ | -0.36355 | -0.39127 | -0.47544 | -1.11237 | -1.00302 |  |  |
| *g*_12_ | -0.03741 | -0.03635 | -0.06974 |  |  |  |  |
| *g*_13_ | -1.37215 | -1.42087 | -1.56281 | -2.59286 | -2.41843 | -0.81479 | -0.81521 |
| *g*_14_ | 0.02696 | 0.02228 |  |  |  |  |  |
| *β*_1_ | 3.89080 | 3.67696 | 3.00550 | 1.55849 | 1.67056 | 9.67903 | 9.73394 |
| *h*_11_ | 0.82715 | 0.85231 | 0.99722 | 1.33258 | 1.27710 | 0.51198 | 0.50982 |
| *h*_12_ | -0.02735 | -0.02713 | -0.07295 | -0.01122 |  |  |  |
| *h*_13_ | 0.50948 | 0.54574 | 0.79229 | 1.31683 | 1.22585 | 0.00383 |  |
| *h*_14_ | 0.00431 |  |  |  |  |  |  |
| R^2^ | 1 | 1 | 1 | 1 | 1 | 0.999998 | 0.999998 |

**Table S10.** Parameter values of *X*_2_ for each iteration using LMA

| Iteration | 1 | 2 | 3 | 4 | 5 | 6 | 7 |
| --- | --- | --- | --- | --- | --- | --- | --- |
| *α*_2_ | 7.37716 | 7.09866 | 8.36585 | 8.36496 | 8.76986 | 7.96230 | 7.90009 |
| *g*_21_ | 0.57390 | 0.61199 | 0.48160 | 0.48148 | 0.47274 | 0.51361 | 0.50829 |
| *g*_22_ | -0.09305 | -0.13992 | 0.03956 | 0.03961 | 0.04588 | -0.01607 |  |
| *g*_23_ | 0.03478 | 0.04854 | 0.00028 |  |  |  |  |
| *g*_24_ | 0.00390 |  |  |  |  |  |  |
| *β*_2_ | 2.27677 | 1.97961 | 3.40254 | 3.40160 | 3.75337 | 2.89325 | 2.90176 |
| *h*_21_ | -0.03079 | -0.05117 | 0.033470 | 0.03328 | 0.04218 |  |  |
| *h*_22_ | 0.81346 | 0.84863 | 0.724340 | 0.72451 | 0.69772 | 0.76145 | 0.76902 |
| *h*_23_ | 0.04719 | 0.05987 | 0.015000 | 0.01474 |  |  |  |
| *h*_24_ | 0.01425 | 0.01472 |  |  |  |  |  |
| R^2^ | 1 | 1 | 1 | 1 | 0.999999 | 0.999999 | 0.999999 |

**Table S11.** Parameter values of *X*_3_ for each iteration using LMA

| Iteration | 1 | 2 | 3 | 4 | 5 | 6 | 7 | 8 |
| --- | --- | --- | --- | --- | --- | --- | --- | --- |
| *α*_3_ | 1.98302 | 2.22799 | 0.99090 | 3.50490 | 3.22457 | 3.22457 | 3.12558 | 4.44033 |
| *g*_31_ | 0.01925 |  |  |  |  |  |  |  |
| *g*_32_ | 0.87489 | 0.84960 | 1.17090 | 0.70573 | 0.72447 | 0.72447 | 0.72239 | 0.50421 |
| *g*_33_ | -0.07383 | -0.06110 | -0.33027 | 0.00858 |  |  |  |  |
| *g*_34_ | -0.05002 | -0.01680 | -0.16062 |  |  |  |  |  |
| *β*_3_ | 3.76784 | 4.17821 | 2.63330 | 5.39371 | 5.12987 | 5.12987 | 4.97183 | 5.25667 |
| *h*_31_ | 0.03747 | 0.01412 |  |  |  |  |  |  |
| *h*_32_ | -0.13402 | -0.10630 | -0.40150 | 0.05275 | 0.01973 | 0.01973 |  |  |
| *h*_33_ | 0.60945 | 0.59233 | 0.67468 | 0.43984 | 0.46477 | 0.46477 | 0.47047 | 0.22851 |
| *h*_34_ | 0.19157 | 0.22665 | 0.19281 | 0.16544 | 0.17779 | 0.17779 | 0.17377 |  |
| R^2^ | 1 | 1 | 1 | 1 | 1 | 1 | 1 | 0.999902 |

**Table S12.** Parameter values of *X*_4_ for each iteration using LMA

| Iteration | 1 | 2 | 3 | 4 | 5 | 6 | 7 | 8 |
| --- | --- | --- | --- | --- | --- | --- | --- | --- |
| *α*_4_ | 0.34430 | 0.22500 | 0.02579 | 0.06440 | -9.8E+15 | -1.5E+07 | 5.16827 | 2.08017 |
| *g*_41_ | 0.78879 | 0.90208 | 1.64417 | 1.33895 | -42.5137 | -9.27070 |  | 0.47096 |
| *g*_42_ | -0.17839 | -0.16099 |  |  |  |  |  |  |
| *g*_43_ | 0.02620 |  |  |  |  |  |  |  |
| *g*_44_ | -0.56299 | -0.77055 | -1.92429 | -1.39245 | 44.07592 | 15.10627 | 0.17173 |  |
| *β*_4_ | 8.21555 | 8.59552 | 9.59250 | 7.43475 | 1.25E+08 | -2.5E+16 | 6.31680 | 5.89774 |
| *h*_41_ | -0.51013 | -0.58854 | -0.90379 | -0.77330 | -10.3405 |  |  |  |
| *h*_42_ | -0.25411 | -0.25044 | -0.09004 |  |  |  |  |  |
| *h*_43_ | 0.25751 | 0.26529 | 0.21429 | 0.09689 |  |  |  |  |
| *h*_44_ | 1.74691 | 1.88053 | 2.37302 | 2.08238 | 16.94432 | 13297138 | 0.27144 | 0.75724 |
| R^2^ | 1 | 1 | 1 | 1 | 0.991864 | 0.985894 | 0.93971 | 0.99997 |
