# Supplementary material for: Identification of a Metabolic Reaction Network from Time-Series Data of Metabolite Concentrations
Source: PLoS One. 2013 Jan 10;8(1):e51212. doi: 10.1371/journal.pone.0051212 (PMC3542379; doi:10.1371/journal.pone.0051212)
Supplement: Information S3 — Evaluation of the performance of our approach in practical application. (DOC) [file pone.0051212.s003.doc]

**Supporting Information S3:**

**Evaluation of the performance of our approach in practical application**

The generic inhibition and activation model was again used to investigate the performance of our approach. To obtain data set close to actual experimental one, noises were allowed to uniformly distribute in the range of 0-5% for the time-series values of each metabolite concentration by perturbing only *X*_1_ (Figure S1). The number of data was varied from 11 points to 51 points. The data fitting was carried out using LOESS.

**
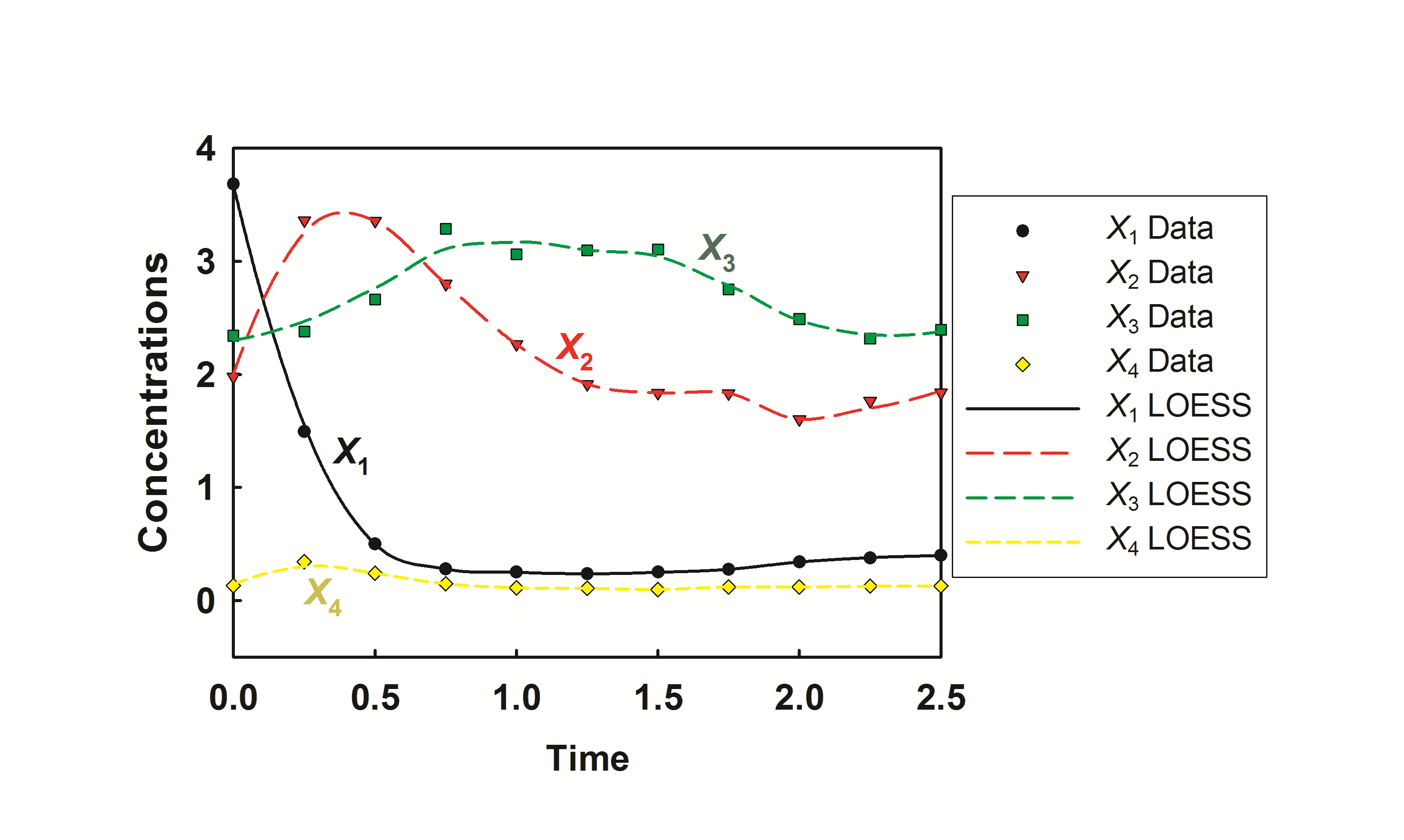
**

**Figure S1.** Information for fitting the generic inhibition and activation model using LOESS (spans of *X*_1_, *X*_2_, *X*_3_ and *X*_4_ are 0.5, 0.5, 0.6 and 0.5, respectively)

The Granger causality was calculated and the result is shown in Table S13.

**Table S13.** Granger causality for the generic inhibition and activation model

|  | X_1_=> | X_2_=> | X_3_=> | X_4_=> |
| --- | --- | --- | --- | --- |
| => X_1_ | N/A | 2.93E-16 | 0.144177 | 4.16E-19 |
| => X_2_ | 4.19E-25 | N/A | 6.36E-08 | 1.01E-08 |
| => X_3_ | 0.008529 | 5.48E-17 | N/A | 2.63E-11 |
| => X_4_ | 1.60E-23 | 5.66E-05 | 0.000271 | N/A |

The criteria mentioned in the main text were set. Parameter estimation and network identification were carried out by LMA with our proposed algorithm. The results are tabulated in Table S14-S17.

**Table S14.** Parameter values of *X*_1_ for each iteration using LMA

| Iteration | 1 | 2 | 3 | 4 | 5 | 6 | 7 |
| --- | --- | --- | --- | --- | --- | --- | --- |
| *α*_1_ | 1.838827 | 9.018346 | 7.409329 | 12.43137 | 13.5826 | 13.08227 | 28.77357 |
| *g*_11_ | -3.16555 | -1.95236 | -2.53834 | -1.30422 | -1.32078 | -0.2531 |  |
| *g*_12_ | 5.221666 | 0.476739 | 1.030395 | 0.329656 | 0.330718 |  |  |
| *g*_13_ | -12.2311 | -4.05971 | -5.18379 | -3.18157 | -3.2898 | -0.93752 | -0.29081 |
| *g*_14_ | -1.95745 |  |  |  |  |  |  |
| *β*_1_ | 0.046956 | 3.770954 | 1.381153 | 5.044655 | 5.494256 | 9.92001 | 26.38361 |
| *h*_11_ | 0.811452 | 0.697652 | 0.805625 | 0.52379 | 0.516954 | 0.307543 | 0.173302 |
| *h*_12_ | 5.7847 | -0.31571 | 0.303687 |  |  |  |  |
| *h*_13_ | -6.46933 | 1.050699 | 0.906927 | 0.09246 |  |  |  |
| *h*_14_ | -2.97301 | 0.283523 |  |  |  |  |  |
| R^2^ | 0.999675 | 0.99947 | 0.999464 | 0.999397 | 0.999398 | 0.999243 | 0.999238 |

**Table S15.** Parameter values of *X*_2_ for each iteration using LMA

| Iteration | 1 | 2 | 3 | 4 | 5 | 6 | 7 |
| --- | --- | --- | --- | --- | --- | --- | --- |
| *α*_2_ | 7.76103 | 0.051163 | 0.409195 | 0.194674 | 0.247795 | 55.97439 | 12.00558 |
| *g*_21_ | 0.478895 | 0.498296 | 0.303459 | 0.85625 | 0.651967 | 0.061798 | 0.272436 |
| *g*_22_ | 10.91739 | 12.15197 | 0.586171 | 0.385059 |  |  |  |
| *g*_23_ | -22.8478 | -19.8586 | 2.66591 | 2.798436 | 3.384467 | 0.060741 |  |
| *g*_24_ | -5.5709 | -6.44479 | -0.29098 |  |  |  |  |
| *β*_2_ | 0.000132 | 0.000326 | 0.502855 | 0.02447 | 0.117628 | 52.74774 | 7.11734 |
| *h*_21_ | -0.43475 | -0.087 |  |  |  |  |  |
| *h*_22_ | 3.526794 | 3.315191 | 0.628913 | 1.643606 | 0.91373 | 0.085594 | 0.385304 |
| *h*_23_ | 4.722317 | 4.892085 | 2.70357 | 3.159092 | 2.833593 |  |  |
| *h*_24_ | -0.18941 |  |  |  |  |  |  |
| R^2^ | 0.983861 | 0.983768 | 0.981473 | 0.979257 | 0.978836 | 0.975905 | 0.973339 |

**Table S16.** Parameter values of *X*_3_ for each iteration using LMA

| Iteration | 1 | 2 | 3 | 4 | 5 | 6 | 7 |
| --- | --- | --- | --- | --- | --- | --- | --- |
| *α*_3_ | 3.42618 | 41.5698 | 0.009716 | 2.739958 | 0.041511 | 0.184325 | 36.81995 |
| *g*_31_ | -0.90366 |  |  |  |  |  |  |
| *g*_32_ | 4.511199 | 1.01979 | 7.175222 | 2.33816 | 3.080551 | 1.622345 | 0.074902 |
| *g*_33_ | -7.54718 | -2.27475 | -7.48054 | -2.5809 |  |  |  |
| *g*_34_ | -1.19637 | 0.293286 | -2.9995 |  |  |  |  |
| *β*_3_ | 2222.58 | 218.8028 | 129810.9 | 3889.947 | 389310.5 | 0.754478 | 37.79896 |
| *h*_31_ | -1.27722 | 0.030817 |  |  |  |  |  |
| *h*_32_ | -2.04479 | -0.90895 | -9.81221 | -5.14884 | -12.5623 | -4.3255 |  |
| *h*_33_ | -0.8466 | -0.17962 | 9.779138 | 5.068093 | 13.64739 | 2.836752 | 0.031468 |
| *h*_34_ | 3.12412 | 1.430963 | 7.446801 | 4.635124 | 9.022561 |  |  |
| R^2^ | 0.95535 | 0.950222 | 0.949709 | 0.948964 | 0.941102 | 0.905236 | 0.889286 |

**Table S17.** Parameter values of *X*_4_ for each iteration using LMA

| Iteration | 1 | 2 | 3 | 4 | 5 | 6 | 7 |
| --- | --- | --- | --- | --- | --- | --- | --- |
| *α*_4_ | 0.003275 | 0.004846 | 0.001477 | 0.001248 | 0.003474 | 8.761753 | 3.663128 |
| *g*_41_ | 1.787642 | 1.828984 | 1.701326 | 1.694169 | 1.640555 | 0.067989 | 0.154481 |
| *g*_42_ | -0.84134 | -1.51279 | 0.333649 |  |  |  |  |
| *g*_43_ | 6.442096 | 7.355161 | 4.871965 | 5.427514 | 4.305068 |  |  |
| *g*_44_ | 0.734926 | 1.119532 |  |  |  |  |  |
| *β*_4_ | 0.000004 | 0.000011 | 0.00001 | 0.000011 | 0.000002 | 10.41001 | 5.227645 |
| *h*_41_ | 0.11387 |  |  |  |  |  |  |
| *h*_42_ | -4.06722 | -4.69139 | -3.89799 | -5.33035 |  |  |  |
| *h*_43_ | 29.99064 | 30.27271 | 28.45857 | 31.72041 | 20.58377 | -0.02351 |  |
| *h*_44_ | 9.630212 | 10.09787 | 9.391017 | 10.59632 | 5.991914 | 0.107342 | 0.252206 |
| R^2^ | 0.992278 | 0.992265 | 0.992184 | 0.991805 | 0.990661 | 0.964904 | 0.963338 |

The predicted network using our proposed algorithm is presented in Figure S2.


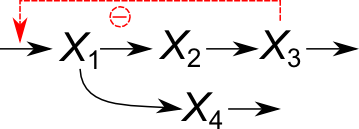


**Figure S2.** Predicted pathway from our approach

The result shows that our approach identifies a metabolic reaction network depending on the data quality regardless of the amount of data. Our approach performs well if the time-series data possess the characteristics of the network. On the other hand, there is a possibility that this approach may not be able to perfectly detail the network because it is difficult to distinguish a network structure from scattered time-series data.
