# Supplementary material for: Identification of a Metabolic Reaction Network from Time-Series Data of Metabolite Concentrations
Source: PLoS One. 2013 Jan 10;8(1):e51212. doi: 10.1371/journal.pone.0051212 (PMC3542379; doi:10.1371/journal.pone.0051212)
Supplement: Information S4 — Additional information for the Lactococcus lactis model. (DOC) [file pone.0051212.s004.doc]

**Supporting Information S4:**

**Additional information for the *Lactococcus lactis* model**

**Table S18.** Information for fitting *Lactococcus lactis* model using LOESS

|  | Glu | G6P | FBP | Lac | Ace |
| --- | --- | --- | --- | --- | --- |
| Piecewise1 | t = 0-50 | t = 0-7 | t = 0-50 | t = 0-50 | t = 0-7 |
| Span1 | 0.1 | 0.9 | 0.1 | 0.4 | 0.7 |
| Piecewise2 |  | t = 8-50 |  |  | t = 8-50 |
| Span2 |  | 0.1 |  |  | 0.9 |

Table S19-S23 tabulates the iterations for parameter values of *X*_1_ to *X*_5_, respectively. The estimation loops were iterated until the most suitable parameters (indicated in red) were found as described in detail in the paper.

**Table S19.** Parameter values of *X*_1_ for each iteration using LMA

| Iteration | 1 | 2 | 3 | 4 | 5 | 6 |
| --- | --- | --- | --- | --- | --- | --- |
| *α*_1_ | 0.002852 | 0.198752 | 0.1585 | 0.141481 | 187.3819 | 20.3529 |
| *β*_1_ | 0.046663 | 0.226706 | 0.19973 | 0.190723 | 190.7807 | 22.27998 |
| *h*_11_ | 0.674177 | 0.897088 | 0.900072 | 0.942731 |  | 0.027577 |
| *h*_12_ | 0.435577 |  |  |  |  |  |
| *h*_13_ | 0.71103 | -0.04374 |  |  |  |  |
| *h*_14_ | -0.50502 | 0.092709 | 0.066249 |  |  |  |
| *h*_15_ | 1.851128 | 1.473718 | 1.501887 | 1.682584 | -0.01043 |  |
| R^2^ | 0.999897 | 0.999719 | 0.99971 | 0.999614 | 0.694423 | 0.886983 |

**Table S20.** Parameter values of *X*_2_ for each iteration using LMA

| Iteration | 1 | 2 | 3 | 4 | 5 |
| --- | --- | --- | --- | --- | --- |
| *α*_2_ | 44.28688 | 6.778078 | 7.984008 | 16.19561 | 2.813854 |
| *g*_21_ | 0.079231 | 0.046891 | 0.031756 | 0.022384 | 0.169215 |
| *g*_22_ | -0.18052 | -0.33919 | -0.31532 |  |  |
| *g*_23_ | 0.349357 | 0.529536 | 0.540671 | 0.247171 | 0.035769 |
| *g*_24_ | 0.216963 | 0.152404 | -0.03522 | -0.02676 | -0.22967 |
| *g*_25_ | -1.4143 | -1.49362 | -0.96623 | -0.7488 | -0.19459 |
| *β*_2_ | 44.49992 | 6.897352 | 7.864257 | 15.83826 | 2.105115 |
| *h*_21_ | 0.069648 |  |  |  |  |
| *h*_22_ | -0.16577 | -0.26368 | -0.26068 | 0.038699 | 0.305156 |
| *h*_23_ | 0.346744 | 0.514913 | 0.529434 | 0.241118 |  |
| *h*_24_ | 0.22253 | 0.183201 |  |  |  |
| *h*_25_ | -1.41732 | -1.50571 | -0.98103 | -0.7546 | -0.1861 |
| R^2^ | 0.999256 | 0.99927 | 0.99926 | 0.999136 | 0.99887 |

| Iteration | 6 | 7 | 8 | 9 | 10 |
| --- | --- | --- | --- | --- | --- |
| *α*_2_ | 2.523076 | 2.045104 | 2.363394 | 0.490795 | 6.135581 |
| *g*_21_ | 0.218247 | 0.259291 | 0.199084 | 0.768696 |  |
| *g*_22_ |  |  |  |  |  |
| *g*_23_ |  |  |  |  |  |
| *g*_24_ | -0.21612 | -0.34528 | -0.31692 |  | -0.23717 |
| *g*_25_ | -0.21449 | 0.129377 |  |  |  |
| *β*_2_ | 1.897926 | 1.293412 | 1.373492 | 0.560201 | 3.484446 |
| *h*_21_ |  |  |  |  |  |
| *h*_22_ | 0.327067 | 0.374313 | 0.328883 | 1.402589 | 0.067234 |
| *h*_23_ |  |  |  |  |  |
| *h*_24_ |  |  |  |  |  |
| *h*_25_ | -0.20487 |  |  |  |  |
| R^2^ | 0.998756 | 0.998713 | 0.998686 | 0.982376 | 0.996531 |

**Table S21.** Parameter values of *X*_3_ for each iteration using LMA

| Iter | 1 | 2 | 3 | 4 | 5 | 6 | 7 | 8 | 9 |
| --- | --- | --- | --- | --- | --- | --- | --- | --- | --- |
| *α*_3_ | 76.04747 | 11.67639 | 47.03347 | 41.90412 | 34.44294 | 521.365 | 431.1458 | 98.90774 | 12.2586 |
| *g*_31_ | -0.76389 | -0.76775 | -1.02388 | -0.92558 | -0.40305 | -0.57846 | 0.007766 |  | 0.423329 |
| *g*_32_ | 1.472218 | 1.475885 | 1.762132 | 2.179366 | 1.71412 | 1.692672 | 1.175224 | 1.138167 |  |
| *g*_33_ | 0.683811 | 0.714087 | 0.523208 |  |  |  |  |  |  |
| *g*_34_ | -0.28989 | -0.43965 |  |  |  |  |  |  |  |
| *g*_35_ | 0.318524 | 0.118386 | -1.28792 | -0.9655 |  |  |  |  |  |
| *β*_3_ | 60.32647 | 1.127247 | 17.17466 | 1.918976 | 10.18136 | 484.29 | 430.3911 | 88.73168 | 12.0847 |
| *h*_31_ | -0.7799 | -0.95366 | -1.13789 | -1.27094 | -0.59146 | -0.58419 |  |  |  |
| *h*_32_ | 1.430425 | 1.017013 | 1.425783 | 1.138414 | 1.30386 | 1.676471 | 1.171732 | 0.993768 | 0.311558 |
| *h*_33_ | 0.747868 | 1.426454 | 1.023747 | 1.532897 | 0.654426 | 0.0277 | 0.005887 | 0.077278 | 0.129582 |
| *h*_34_ | -0.2416 |  |  |  |  |  |  |  |  |
| *h*_35_ | 0.237418 | -0.72029 | -1.59223 | -1.89319 | -0.46811 |  |  |  |  |
| R^2^ | 0.998942 | 0.998899 | 0.998921 | 0.998898 | 0.997608 | 0.988568 | 0.974871 | 0.948604 | 0.894269 |

**Table S22.** Parameter values of *X*_4_ for each iteration using LMA

| Iteration | 1 | 2 | 3 | 4 |
| --- | --- | --- | --- | --- |
| *α*_4_ | 12.02546 | 0.670717 | 1.038449 | 0.23796 |
| *g*_41_ |  |  |  |  |
| *g*_42_ | 3.116522 | 1.146196 | 1.360695 |  |
| *g*_43_ | -1.90215 | 0.199349 |  | 0.84994 |
| *g*_44_ | 0.912457 |  |  |  |
| *g*_45_ |  |  |  |  |
| R^2^ | 0.994705 | 0.981629 | 0.978498 | 0.937133 |

**Table S23.** Parameter values of *X*_5_ for each iteration using LMA

| Iteration | 1 | 2 | 3 | 4 |
| --- | --- | --- | --- | --- |
| *α*_5_ | 0.272811 | 0.215599 | 0.33434 | 0.092744 |
| *g*_51_ |  |  |  |  |
| *g*_52_ | 0.524291 | 0.324206 | 0.492909 |  |
| *g*_53_ | 0.019338 | 0.183173 |  | 0.52135 |
| *g*_54_ |  |  |  |  |
| *g*_55_ | 0.171962 |  |  |  |
| R^2^ | 0.920531 | 0.919822 | 0.916616 | 0.908611 |

**Table S24.** Parameter and initial values for *Lactococcus lactis* model

| **Parameters** | | **Parameters** | | **Parameters** | | **Parameters** | | **Initial values** | |
| --- | --- | --- | --- | --- | --- | --- | --- | --- | --- |
| Y_1_ | 0.141481 | Y_6_ | 0.190726 | Y_11_ | 0.199084 | Y_16_ | 0.077278 | *X*_1_ | 20 |
| Y_2_ | 2.363394 | Y_7_ | 1.373492 | Y_12_ | -0.31692 | Y_17_ | 1.146196 | *X*_2_ | 0.4 |
| Y_3_ | 98.90774 | Y_8_ | 88.73168 | Y_13_ | 0.328883 | Y_18_ | 0.199349 | *X*_3_ | 0.4 |
| Y_4_ | 0.670717 | Y_9_ | 0.942731 | Y_14_ | 1.138167 | Y_19_ | 0.324206 | *X*_4_ | 0.1 |
| Y_5_ | 0.215599 | Y_10_ | 1.682584 | Y_15_ | 0.993768 | Y_20_ | 0.183173 | *X*_5_ | 0.3 |
